# Heterosubtypic T-Cell Immunity to Influenza in Humans: Challenges for Universal T-Cell Influenza Vaccines

**DOI:** 10.3389/fimmu.2016.00195

**Published:** 2016-05-19

**Authors:** Saranya Sridhar

**Affiliations:** ^1^Jenner Institute, University of Oxford, Oxford, UK

**Keywords:** influenza, T-cells, universal vaccine, pandemic influenza, heterosubtypic immunity, correlates of protection

## Abstract

Influenza A virus (IAV) remains a significant global health issue causing annual epidemics, pandemics, and sporadic human infections with highly pathogenic avian or swine influenza viruses. Current inactivated and live vaccines are the mainstay of the public health response to influenza, although vaccine efficacy is lower against antigenically distinct viral strains. The first pandemic of the twenty-first century underlined the urgent need to develop new vaccines capable of protecting against a broad range of influenza strains. Such “universal” influenza vaccines are based on the idea of heterosubtypic immunity, wherein immune responses to epitopes conserved across IAV strains can confer protection against subsequent infection and disease. T-cells recognizing conserved antigens are a key contributor in reducing viral load and limiting disease severity during heterosubtypic infection in animal models. Recent studies undertaken during the 2009 H1N1 pandemic provided key insights into the role of cross-reactive T-cells in mediating heterosubtypic protection in humans. This review focuses on human influenza to discuss the epidemiological observations that underpin cross-protective immunity, the role of T-cells as key players in mediating heterosubtypic immunity including recent data from natural history cohort studies and the ongoing clinical development of T-cell-inducing universal influenza vaccines. The challenges and knowledge gaps for developing vaccines to generate long-lived protective T-cell responses is discussed.

## Introduction

Influenza is a major public health problem with annual influenza epidemics which affect an estimated 15% of the global population (1) that are punctuated by pandemics capable of causing more severe morbidity and mortality (2). Host protection against influenza infection is primarily mediated by neutralizing antibodies against the viral surface glycoproteins, hemagglutinin (HA) and neuraminidase (NA). These annual epidemics and periodic pandemics are a result of constant antigenic variation in an evolving influenza virus attempting to evade host protective immunity. New strains arise through the gradual accumulation of point mutations in the viral surface glycoproteins (antigenic drift) or genetic reassortments between different viral subtypes (antigenic shift) with the potential to cause influenza epidemics and pandemics, respectively.

Vaccination remains the most cost-effective strategy against influenza and has been in use since the 1940s. However, a major limitation of current vaccines, both inactivated and live attenuated vaccines, is the inability to provide high levels of protective efficacy in the face of this antigenic variation. Current vaccines that offer high levels of strain-specific protection are less effective against the unpredictable emergence of new antigenically variant strains, not represented in the vaccine (3, 4). This lack of efficacy against mismatched strains has two key consequences: one, it necessitates yearly reformulation, production, and deployment of influenza vaccines based on the prediction of strains that may circulate in the subsequent influenza season; two, in the event of the emergence of a completely novel reassortant virus, there is little or no efficacy leaving individuals at high risk of infection with potential for pandemic spread. The first pandemic of the twenty-first century in 2009, zoonotic human infections with highly pathogenic avian and swine influenza viruses and the difficulty in predicting antigenic variation, underscores the need to develop new influenza vaccines capable of mediating protection against a broad range of influenza strains. The basis of such broadly protective “universal influenza vaccines” is the phenomenon of heterosubtypic immunity, wherein host immune responses targeted toward viral epitopes highly conserved across all influenza subtypes confers protection against infection and disease. Heterosubtypic immunity in animal models has historically been shown to be predominantly mediated by CD8^+^ T-cells, although evidence in humans was limited. This review focuses on the recent studies undertaken during the 2009 H1N1 pandemic that provided the evidence in humans for T-cell-mediated heterosubtypic immunity (5, 6). The distinctive roles of the four broad subsets of memory T-cells – effector memory (T_EM_), central-memory (T_CM_), effector memory RA (T_EMRA_), and more recently, tissue-resident memory (T_RM_) – with different phenotypes, transcriptional signatures, anatomical location, and migration patterns (7, 8) in heterosubtypic immunity is discussed. I argue that a major barrier to develop T-cell-inducing vaccines for influenza remains in our understanding of the key determinants in positioning protective T-cells in the respiratory tract to limit viral replication and deliberate on some of the immunological challenges faced in developing T-cell-inducing influenza vaccines.

## Antibody-Mediated Immunity

The role of antibodies in protective immunity has traditionally been limited to homosubtypic rather than heterosubtypic immunity. However, theoretically, antibodies recognizing conserved epitopes could confer heterosubtypic immunity. There has been renewed interest in antibody-mediated heterosubtypic immunity through broadly reactive monoclonal antibodies (9), antibodies directed toward the highly conserved HA stalk domain (10), and non-neutralizing antibodies. Cross-reactive non-neutralizing antibodies that mediate antibody-dependent cellular cytotoxicity have been shown to confer heterosubtypic protection in mice (11) and non-human primates (NHPs) (12), and are detectable in humans in the absence of neutralizing antibodies (13, 14). New promising vaccine strategies targeting antibodies to the conserved stalk region of HA using “headless” HA (15) can confer heterosubtypic protection in mice (16) and ferrets (17) and are detectable in young and older adults (18). Another conserved antigenic target is the Matrix 2 ectodomain, with antibodies to this region capable of providing heterosubtypic protection in animal models (19). However, the role of these antibodies in protecting individuals in the context of a pandemic remains to be understood, and there is some evidence for a disease-enhancing role for these antibodies (20). Therefore, while broadly cross-reactive antibodies are an exciting prospect for vaccine development, further development and cautious evaluation may be necessary.

## Heterosubtypic Immunity in Animal Models

The phenomenon of heterosubtypic immunity was elegantly demonstrated by Schulman and Kilbourne in a murine model of influenza infection. In mice infected with a H1N1 strain and subsequently challenged with a lethal H2N2 virus, they observed a reduction in viral titers in the lung, lung pathology, and mortality (21). They remarked on the early reduction in viral titers despite an absence of antibodies, suggesting that protection may not have been mediated by neutralizing antibodies. Since then, studies in multiple animal models including NHPs have reproduced this observation (22–25) noting reduction in viral load, lung pathology, weight loss, and mortality, but not infection. Investigation of the immunological mechanisms in animal models revealed the role of CD8^+^ T-cells and CD4^+^ T-cells in mediating this cross-protective immunity (26, 27).

Cytotoxic CD8^+^ T-cells are thought to be the primary mediators of heterosubtypic immunity in animal models. Early work demonstrated that CD8^+^ T-cells, both polyclonal and those grown from cloned cell lines, were cross-reactive for different type A influenza viruses recognizing conserved peptides predominantly from the internal viral proteins (28–31). The necessity for CD8^+^ T-cells in mediating protection against a primary infection was demonstrated in adoptive transfer experiments and by observing delayed viral clearance and increased mortality in CD8^+^ T-cell deficient transgenic mice (32, 33). In mice primed by infection, preexisting memory CD8^+^ T-cells established following the first infection mediated enhanced viral clearance and reduced pathology against subsequent heterosubtypic challenge (34–36). However, more recent work suggests that CD8^+^ T-cells may work in concert with non-neutralizing antibodies and alveolar macrophages in mediating protection against a lethal virus challenge (11, 37).

The effector mechanisms used by CD8^+^ T-cells to mediate the killing of virus-infected cells occur mainly through contact-dependent release of cytotoxic perforin or granzyme granules, apoptosis triggered by Fas/FasL interaction (38), and antiviral suppressor function of secreted IFN-γ and other cytokines and chemokines (39–41). Recently, a subset of non-cytolytic CD8^+^ T-cell-secreting IL-17 (Tc17) generated *in vitro* has been shown to mediate protection against lethal influenza through an IFN-γ-dependent mechanism (42).

The role of CD4^+^ T-cells in mediating heterosubtypic immunity is less clear but is an increasing focus of attention and is reviewed elsewhere (43). Although adoptive transfer of influenza-specific CD4^+^ T-cells demonstrate the ability of CD4^+^ T-cells to mediate protection, recent work transferring physiological frequencies of CD4^+^ T-cells specific for a single influenza epitope resulted in little protection against subsequent influenza challenge (44). Nevertheless, there is mounting evidence of CD4^+^ T-cells facilitating heterosubtypic immunity through different mechanisms including direct cytolytic activity and interactions with B cells, or CD8^+^ T-cells (45–47).

## Epidemiological Clues of Heterosubtypic Protection in Humans

Is there any evidence in human populations that natural heterosubtypic immunity can limit disease severity? To demonstrate heterosubtypic immunity in humans requires the recording of the clinical outcomes of individuals previously infected with influenza as they encounter a new antigenically distinct strain. A few opportunistic studies undertaken when new pandemic strains had emerged provide epidemiological evidence for natural heterosubtypic immunity. The first report by Slepushkin followed adults as the new H2N2 pandemic strain emerged in 1957 (48). Over three influenza waves in 1957 – a spring seasonal H1N1 influenza wave, a summer pandemic H2N2 wave, and a second pandemic H2N2 wave in the fall – the rates of influenza-like-illness (ILI), but not laboratory-confirmed influenza, were recorded in adults. Two key observations were made. First, individuals who reported an ILI during the spring seasonal H1N1 influenza wave were less likely to have ILI through the H2N2 summer pandemic wave ~2 months later and during the fall wave ~5 months later. Second, the level of cross-protection to pandemic H2N2 was short-lived, declining but not abrogated, within 3–5 months after seasonal H1N1 influenza infection. Although laboratory-confirmed influenza was not recorded, this seems to be the first evidence that previous seasonal influenza infection conferred protection against an antigenically distinct pandemic influenza strain.

Epstein extended these observations using historical data of laboratory-confirmed influenza among participants in the Cleveland family study during the 1957 H2N2 pandemic (49). Adults with laboratory-confirmed H1N1 influenza between 1950 and 1957 were ~3 times less likely to have symptomatic laboratory-confirmed pandemic H2N2 influenza compared to those who were not previously infected. A particularly interesting finding was the absence of any neutralizing antibodies to the pandemic H2N2 virus in these participants prior to onset of the pandemic, suggesting alternatives to neutralizing anti-HA antibodies as immune correlates of heterosubtypic protection. However, the duration between the last seasonal influenza infection and exposure to the new H2N2 strain was not known, which would have enabled determination of durability of this cross-protection. Similar observations of a lowered risk of influenza illness in those with previous infections was observed in Japanese school children during the re-emergence of H1N1 in 1977–1978 (50) and, more recently, during the 2009 H1N1 pandemic in children in Hong Kong (51). These studies show that infection generates immune responses, most likely not neutralizing antibodies, which confer cross-protective immunity against development of symptomatic influenza in humans.

However, there remain a number of unanswered questions. How long does this natural cross-protective immunity last in the population? Data from the 2009 pandemic suggest that protection lasts at least 1 year after previous seasonal influenza infection (51), although an optimistic reading of the data collected by Epstein during the 1957 pandemic may suggest more durable cross-protective immunity. How does age, number of previous infections and severity of infections, viral load, and ethnicity impact this cross-protective immunity? None of the studies, to date, have demonstrated whether this cross-protection reduces the risk of severe disease and death and if so, in what proportion of the population? This is particularly important in order to define clinical end-points that can be measured when evaluating efficacy of candidate universal influenza vaccines. The epidemiological evidence that natural cross-protective immunity can reduce the risk of severe disease, hospitalizations, death, or decrease ongoing transmission remains to be found. Regardless, this partial and potentially short-lived protection offers a template for the development of broadly protective influenza vaccines. The key lies in elucidating the immunological mechanisms of natural heterosubtypic immunity in humans and developing vaccines that improve on nature.

## T-Cells and Heterosubtypic Immunity in Humans

### T-Cells Do Not Distinguish between Influenza Subtypes

Peripheral blood mononuclear cells (PBMCs) from human donors stimulated *in vitro* with influenza A virus (IAV) do not lyse influenza B virus-infected target cells but are capable of lysing target cells infected with a different IAV subtype by targeting cross-reactive antigens (52–54). More recently, similar polyclonal CD8^+^ and CD4^+^ T cell cross-reactivity to IAV strains to which individuals were not previously exposed has been demonstrated with human H1N1 (55–59), H3N2 (60), H3N2v (61), H2N2 (60), and avian H5N1 (62, 63) and H7N9 (64, 65) viruses. Fundamental to the development of vaccines exploiting the ability of T-cells to recognize variant influenza viruses is the identification of antigenic targets of these cross-reactive T-cells and evidence that cross-reactive T-cells mediate protection in humans.

### Specificity of Cross-reactive T-Cells in Humans

The major antigenic targets of these cross-reactive T-cells are epitopes in the highly conserved internal proteins of influenza, particularly polymerase-binding protein (PB) 1, matrix 1 (M1), and nucleoprotein (NP). In early experiments, Gotch and colleagues used recombinant vaccinia virus to express each viral protein and infected autologous target cells to determine the main antigenic targets of cytotoxic T-cells (CTLs) from human donors. In a small sample of six donors they found M1, NP, and PB2 to be the most commonly recognized proteins with recognition restricted by HLA-type (66). These findings have been replicated by more recent studies. Assarsson et al. identified a set of 54 epitopes conserved across human and avian influenza virus A subtypes and strains, the majority from the internal PB1, M1, and NP proteins, recognized by PBMCs from human donors (67). These class I- and class II-restricted epitopes covered six HLA class I supertypes (A1, A2, A3, A24, B7, and B44) and a single HLA class II supertype (DR) to estimate >90% coverage across populations. Interestingly, among 20 random HLA untyped human donors, 75% responded to the pool of the class I-restricted epitopes while 80% responded to the pool of class II-restricted epitopes. Immunoinformatic analysis of the literature of all the published CD8^+^ and CD4^+^ conserved T cell epitopes presented by a variety of HLA class I and class II molecules (www.iedb.org) revealed that these epitopes were most commonly found in the internal viral proteins (68). This immunodominance of M1, NP, and PB1 was also observed while mapping responses of CD4^+^ and CD8^+^ T-cells to the entire genome of an H5N1 virus in unexposed individuals (62). Notably, in addition to the high proportion of responders, frequencies of T-cell responses are also highest to these antigens (62). Not all these conserved epitopes are necessarily protective and no studies identifying protective epitopes in humans have been conducted. One experimental challenge study mapped responses to the overlapping peptides pools covering the entire length of NP and M1 proteins. In this study, reduced disease severity was associated with higher frequencies of CD4 T-cells specific for entire length of NP and M1 proteins rather than responses to individual epitope(s) (69).

### Evidence for T-Cell-Mediated Cross-Protective Immunity in Humans

Despite the wealth of evidence for both CD4^+^ and CD8^+^ T-cell-mediated cross-protection in animals, human studies integrating immunological analysis with well-defined clinical outcomes are scarce. The first experimental evidence was presented by McMichael and colleagues who used a human experimental challenge model which involved nasal inoculation of individuals with high doses of a lab-adapted attenuated H1N1 virus strain. In individuals born after 1968 who had never been exposed to the H1N1 subtype, the frequency of MHC class I-restricted CTLs prior to infection was associated with decreased viral shedding, but not lower symptom severity (70). This was the first evidence in humans for the potential of cross-reactive CD8^+^ T-cells to mediate protection when antibodies failed to prevent infection by a new virus strain. More recently, this study was repeated using a seasonal H3N2 or H1N1 viruses to infect individuals lacking neutralizing antibodies to the infecting virus strain. In contrast to the challenge study by McMichael et al., this study found higher frequencies of peripherally circulating IFN-γ-secreting CD4^+^, rather than CD8^+^ T-cells, recognizing peptides from NP and M1 correlated with less viral shedding and reduced symptom severity (69). Despite these two studies, the question remained as to whether in the context of natural community-acquired influenza infection, rather than experimental challenge, cross-reactive T-cells were associated with protection.

Two separate studies undertaken as the 2009 pandemic H1N1 (pH1N1) virus strain emerged have shed light on this hitherto unresolved question. In a large cohort of pH1N1 seronegative adults recruited and followed during the evolving pandemic, individuals with higher frequencies of preexisting cross-reactive CD8^+^IFN-γ^+^IL-2^−^ T-cells recognizing highly conserved epitopes in the PB1, M1, and NP internal core proteins of influenza A had milder symptoms and lower risk of viral shedding following pandemic influenza infection (6). Further characterization of these cross-protective CD8^+^T-cells identified the CD45RA^+^CCR7^−^ late-effector subset of memory T-cells that correlated with protection against symptomatic illness. Although this study did not find an association of protection with CD4^+^ T-cells, the role of CD4^+^ T-cells could not be ruled out. These CD8^+^ T-cells were not generated by inactivated vaccines, and therefore, it was apparent that previous seasonal influenza infection induced and maintained this protective T-cell pool (57). This was the first evidence for CD8^+^ T-cell-mediated cross-protection against community-acquired natural influenza infection in humans.

These findings were subsequently corroborated by a second study, which found NP-specific T-cell-secreting IFN-γ (not separated into CD4^+^ and CD8^+^) were associated with a decreased risk of viral shedding but not symptom severity, following pandemic influenza infection (5). Both studies found CD8^+^ T-cells did not reduce risk of infection while severe disease, hospitalizations, or death were not among the clinical outcomes recorded in these studies. Thus, the role of cross-reactive T-cells in protecting against severe disease is less clear. One study found hospitalized patients with severe H7N9 influenza who recovered earlier had a more rapid induction of CD8^+^IFN-γ^+^ responses compared to those with a longer stay (71), but it is not clear how this finding might be confounded by viral determinants of severe disease. The opportunistic natural history cohort studies show that cross-reactive CD8^+^ T-cells can reduce viral shedding and ameliorate mild illness albeit whether they mediate protection against severe disease, hospitalization, death, and household transmission remains to be determined (Table 1).

**Table 1:** **Key human studies of evidence of T-cell-mediated protection against influenza**.

Reference	Study population	Study design	Infecting strain				Cellular responses				Protection against			
			IAV infection	Strain	Previous exposure	NtAb negative	CD4	CD8	T-cell correlate	Antigenic targets	Infection	Symptom severity	Shedding	Severe disease/mortality
McMichael et al. (70)	Adults	Prospective cohort	Experimental challenge	H1N1	No	Yes	ND	+	CD8^+^ cytotoxicity	Epitopes in NP and M1	No	No	Yes	ND
Wilkinson et al. (69)	Adults	Prospective cohort	Experimental challenge	sH1N1 and sH3N2	Yes	Yes	+	−	CD4^+^IFN-γ^+^	NP and M1	No	Yes	Yes	ND
Sridhar et al. (6)	Adults seronegative to pH1N1	Prospective cohort study	Natural community-acquired infection	pH1N1 2009	No	Yes	No	+	CD8^+^IFN-γ^+^IL-2^−^CD45RA^+^CCR7^−^	Conserved epitopes in NP, M1, and PB1	No	Yes	Yes	ND
Hayward et al. (5)	Adults and children	Prospective cohort study	Natural community-acquired infection	sH1N1, sH3N2, and pH1N1 2009	Yes	No	ND	ND	CD3^+^IFN-γ^+^	NP	No	No	Yes	ND
Wang et al. (71)	Adults hospitalized with H7N9 infection	Hospital-based study	Natural community-acquired infection	H7N9	–	–	−	+	CD3^+^CD8^+^IFN-γ^+^	ND	No	No	No	Shorter hospital stay

*ND, not determined*.

## Mucosal Immune Responses are Essential in Influenza Protection

There is now increasing consensus on the importance of T-cells being present locally in the airway or parenchyma of the respiratory tract to protect against influenza. The presence of protective immune responses in the lung is particularly important as severe influenza is due to lung infection, and highly pathogenic avian influenza viruses (e.g., H5N1) have tropism for the lower respiratory tract. In animal models, cross-protection is associated with capacity of CD8^+^ T-cells to home to the lung (72, 73), expression of the integrin VLA-1, which regulates homing to respiratory mucosa (74) and CD8^+^ T-cells in the airways (74). Pulmonary T_RM_ cells, which are located in the airways, have greater protective capacity than circulating memory CD8 T cells (75). The protective CTL populations that develop during IAV infection include T_RM_ cells, which are embedded in the walls of the airways, as well as cells shed into the lumen, which can be collected by bronchoalveolar lavage. Recent studies have shown that deposition of CTLs into the lumen of airways is sufficient to provide protection in a transfer model (76). Influenza-specific memory CD8^+^ and CD4^+^ T-cells are abundantly found following bronchoalveolar lavage of infected patients and healthy adults, although they have a much higher activation threshold compared to peripherally circulating antigen-specific memory T-cells (77–79).

A subset of memory T-cells, T_RM_ that do not circulate and are restricted to peripheral tissues have been described (80). Lung T_RM_ cells are induced following antigen exposure in the lung, require CD4^+^ T-cell help for functional development (81), and persistent antigen exposure for long-term maintenance (82). They are characterized by the surface expression of CD69 and CD103 surface molecules (83), can rapidly trigger an antiviral state (84, 85), and have been shown to be essential and sufficient in mediating heterosubtypic protection against lethal influenza in murine models (75, 86). In rhesus monkeys infected with IAV, NP-specific T_RM_ cells were found in the lung in the absence of a robust peripheral memory T-cell response (87). Human lung CD69^+^CD103^+^ T_RM_ cells differ in many respects from those present in peripheral blood and are enriched for memory cells able to respond to common respiratory pathogens, such as influenza virus (80, 88). These cells have been shown to recognize and kill influenza-infected epithelial cells, suggesting that they could be providing rapid antigen-specific response to influenza infection before the influx of circulating memory T-cells (89). However, direct proof that T_RM_ cells are associated with reduced disease severity in humans is very difficult to demonstrate as lung tissue, obtained from lung resections or autopsy samples, is needed to measure T_RM_ cells.

## T-Cell-Based Universal Vaccine Development

Efforts to develop influenza vaccines capable of conferring protection against a broad spectrum of virus strains is founded on this immunological evidence base for T-cell-mediated heterosubtypic immunity in humans and animal models. It is worthwhile to note that such vaccines are designed to reduce illness severity rather than prevent infection. In contrast to current influenza vaccines targeted to prevent infection, the evaluation, licensure, and deployment of a vaccine not designed to prevent infection but to limit symptoms will be challenging. A number of candidate vaccines, the majority expressing NP and M1 antigens through different vaccine delivery platforms, are in different stages of clinical development. The leading candidate is a modified vaccinia Ankara (MVA) viral vector encoding NP and M1 proteins, MVA-NP + M1, which has shown safety in phase I trials and induces NP- and M1-specific CD8^+^IFN-γ^+^ T cells (90). In a small phase IIa experimental challenge study, this vaccine showed reduction in the duration of viral shedding (91). Another approach using highly conserved multi-epitope small peptides has shown induction of CD8^+^ T-cells in a phase I trial (92), but this vaccine has failed to reduce viral load or symptom score in a phase IIa challenge study (93). Another such candidate is Multimeric-001, a recombinant protein that contains conserved linear epitopes from the NP, M1, and HA proteins of IAVs that can induce T-cells when given to elderly adults in phase I and II trials (94). The licensed live attenuated influenza vaccine has been shown to induce cross-reactive cellular immune responses (95) and studies have demonstrated a moderate level of protection against mismatched antigenically distinct strains (96). However, there are less data available on whether the vaccine is protective against a pandemic strain and can reduce symptom severity in infected individuals. Other experimental approaches including virus-like particles, RNA replicons, DNA vaccines, and adjuvants continue to be developed and are in phase I clinical trials (Table 2).

**Table 2:** **Universal T-cell vaccine candidates in clinical trials**.

Type of vaccine	Name	Description	Influenza antigens	Adjuvant	Clinical phase	Reference
Fusion protein	Multimer-001	Fusion protein of multiple linear epitopes	HA, NP, and M1 (IAV and IBV)	Montanide ISA 51VG	Phase I	(94)
Peptide-based	FLU-v	Polypeptide mixture	M1, M2, and NP	ISA-51	Phase IIa	(92, 93)
Peptide-based	FP01	Nanoparticle vaccine of six peptides	Internal conserved proteins	None	Phase I
Viral vector	MVA-NP + M1	Modified vaccinia Ankara vector	NP + M1 from strain A/Panama/2007/99	None	Phase IIa	(91)
Viral vector	ChAdOX1-NP + M1	Chimpanzee adenovirus	NP + M1 from strain A/Panama/2007/99	None	Phase I	(97)
Virus-like particles		VLP using recombinant baculovirus	HA, NA, and M1	ISCOMATRIX	Phase I	(98)

## Challenges in Developing a T-Cell Vaccine for Influenza

### Generating Durable Memory in the Respiratory Tract

Successful vaccination requires the generation of durable antigen-specific memory responses, which, for acute respiratory infections like influenza, must be capable of rapid recall to the respiratory tract. Different subsets of memory CD8^+^ T-cells, with different phenotypes, transcriptional signatures, anatomical location, migration patterns, and effector functions (7, 8) develop following the survival of a small proportion of CD8^+^ T-cells generated during the primary effector response. Animal models have informed our understanding of the three signals, namely, antigen (signal 1), costimulation (signal 2), and proinflammatory environment (signal 3) during primary infection that determines subsequent development of effector and memory T-cell responses (99). Antigen persistence was shown to maintain memory T-cells in the lung and draining lymph nodes (100) but was not necessary to maintain peripheral memory T-cells capable of airway homing (101). The critical role of respiratory dendritic cells (RDCs), which sample and present antigen in directing the differential development of effector and memory CD8^+^ T-cells in local and peripheral sites, is becoming evident. CD103^+^-migratory RDCs support the development of lung homing effector CD8^+^ T-cells while CD103^−^ dendritic cells support generation of CD8^+^ T_CM_ that remain in the local lymph nodes (102, 103). Chemokine production, particularly CCR5 and CXCR3, are important in regulating contraction and generation of memory T-cells following influenza infection (104, 105). As our understanding of these mechanisms grows, rational design of vaccination strategies to target specific cells or pathways may become more feasible. For example, vaccination using antigen coupled to antibodies targeted exclusively to respiratory DCs can induce T_RM_ cells in the lung and confer protection against influenza challenge (106). The use of adjuvants that limit IL-12-enhanced CXCR3 expression results in better protection and increased CD8^+^ memory T-cells to the airways (107). Another strategy that exploits this migrational imprinting of memory T-cells to mucosal sites is called “prime and pull,” where chemokines to mucosal sites pulled effector CD8 T-cells to differentiate into T_RM_ (108). This may be particularly relevant for vaccination of adults and elders who are already primed and have existing memory T-cells from previous exposures but may not be applicable in infants or young children in whom a significant memory pool may not yet be established.

The route of delivery is crucial in generating local and systemic immune responses. Mucosal vaccination delivering antigen to the respiratory tract generates both local and systemic immune responses comparable to parenteral administration (109). The licensed intranasal live influenza vaccine induces systemic and mucosal cross-reactive CD8^+^ and CD4^+^ T-cells (95, 110). However, what remains unclear is whether intranasal delivery generates memory T-cells in the lung. Deliberate upper or lower respiratory tract vaccination in mouse and macaques suggests that localized nasal infection does not stimulate protective lung immunity (111, 112), although whether the upper and lower respiratory tract are similarly disconnected in humans is a contentious issue. Aerosol delivery of measles vaccine (113), BCG (114), and an MVA viral-vectored vaccine have shown the feasibility of this approach and found preferential induction of lung over peripheral T-cell responses (115). However, the most optimal strategy would be to harness both local and systemic immunity, and data from tuberculosis vaccination seem to suggest that simultaneous mucosal and parenteral administration may be a promising avenue to explore (116). A recent study has shown that combining systemic and mucosal vaccination using an adenoviral vector expressing NP conferred heterosubtypic protection in mice 8 months after vaccination (117).

In addition to the persistence of antigen, repetitive antigen exposure also influences memory T-cell development. Murine models of multiple antigen exposure reveal changing expression levels of transcription factors in memory CD8^+^ T-cells, decreased proliferation capacity, preferential diminution of antigen-specific memory CD8^+^ T-cell-secreting IL-2 without a concomitant decrease in IFN-γ secretion, and a movement of memory cells to non-lymphoid compartments including the blood and peripheral organs (118). This would suggest that recurrent acute infections skew the development of antigen-experienced memory T-cells toward an activated IFN-γ-only-secreting, circulating or mucosal, T-cell phenotype that is primed to protect against inevitable subsequent infections. It is tempting to speculate that the higher frequencies of CD8^+^CD45RA^+^CCR7^−^ late-effector T-cells associated with protection against natural infection in humans (6) may be a result of multiple natural influenza infections. If multiple antigen exposures do skew memory T-cells to a more protective phenotype, prime-boost vaccination may be a more promising strategy.

One significant challenge is to maintain durable memory T-cell responses following vaccination. In humans, long-lived memory T-cells are detectable decades after a single smallpox (119) vaccination or pulmonary hantavirus infection (120). However, influenza-specific memory T-cells in the blood seem to be less durable declining rapidly within a few months after a single infection (121, 122) with a suggested half-life of up to 2–3 years (123). T-cell responses, strain-specific but not cross-reactive, have been observed in children lasting up to 1 year after live vaccination (124). The detection of influenza-specific lung T_RM_ cells in older adults (80) and antigen-specific memory T-cells in the blood up to 15 years after infection (125), most likely due to multiple infections, offers some optimism for generating long-lived cellular immunity to influenza.

### Covering Diverse Populations and Limiting T-Cell Escape

A significant challenge to developing a T-cell inducing vaccine for widespread use would be to provide sufficient coverage to individuals of diverse HLA haplotypes. Recent work suggests that some ethnicities with particular HLA alleles may have limited CD8^+^ T-cell responses even to conserved epitopes in NP and M1 (64) and have different clinical outcomes of influenza infection (126). These studies highlight the need to examine T-cell responses in the context of ethnicity. Indeed, there are limited data on the magnitude, quality, or breadth of influenza-specific T-cell responses in African, Latin American, or Asian populations.

The identification of naturally occurring CD8^+^ T-cell-mediated mutations in viral NP that resulted in immune escape and replacement of the wild-type sequence raises the theoretical concern for vaccine-mediated T-cell escape (127, 128). This selection pressure is likely to be maximally targeted toward the very same immunodominant epitopes that are critical in conferring protective T-cell immunity. One strategy to circumvent this problem is to vaccinate using a range of mutants of immunodominant epitopes that seems to generate memory T-cells against the wild-type dominant sequence (129). However, the likelihood of such T-cell escape as observed with HIV and HCV is less likely for influenza given the presence of numerous epitopes that remain unchanged through viral evolution. Indeed, modeling population-level deployment of an effective cross-protective vaccine revealed the high likelihood of such a strategy slowing down the emergence of antigenically distinct influenza strains (130).

### Rebalancing Protection over Pathology

A significant challenge in developing T-cell vaccines is the need to balance the induction of sufficient quality and quantity of CTLs against the possibility of T-cells contributing to immunopathology. In humans, it is likely that virus-induced inflammation plays a major role in lung pathology, and therefore, rapid viral clearance would be critical in limiting lung pathology. However, the increasing evidence that effective killing of virus-infected cells by CD8^+^ T-cells in the lung is accompanied by non-specific tissue destruction warrants greater understanding, particularly for vaccines seeking to induce large numbers of effector T-cells in the lung. The role of T-cells in causing lung pathology during infection is reviewed in detail elsewhere (131). Briefly, the role of T-cell immunopathology in influenza has been best demonstrated in animal models delinking viral replication-induced inflammation from T-cell induced inflammation. In these models, it has been demonstrated that lung pathology is caused by pro-inflammatory T-cell secreted IFN-γ/TNF-α and T-cell cytolysed virus-infected cells releasing pro-inflammatory chemokines and cytokines prior to cell death. In humans, delineating the role of T-cells in lung injury during viral infection is difficult. One study did report higher frequencies of peripheral and respiratory virus-specific CD4^+^ T-cells post-infection in severe influenza (132), although, whether T-cells caused severe disease or if this was a result of uncontrolled viral replication is difficult to distinguish. Some case reports of lung injury following influenza vaccination have been reported, but whether vaccination caused lung pathology or the mechanisms of injury is not clear (133, 134). Vaccine delivery by aerosol is gaining traction, but theoretical concerns about T-cell induced lung pathology, particularly in older age groups with preexisting influenza-specific T-cells in the respiratory tract, would need to be assuaged. Multiple immunomodulatory mechanisms have been identified to dampen the effector T-cell response including anti-inflammatory cytokines/chemokines, regulatory T-cells, and costimulatory/inhibitory surface molecules. Strategies exploiting such immunomodulatory mechanisms to shift the balance toward protection rather than pathology include use of costimulatory molecules as adjuvants, peptide-tolerization, and targeting the vaccine to particular cell types (135, 136).

### Identifying a Correlate of Protection

Vaccine development is immeasurably advanced if a correlate of protection can be identified and validated for use in humans. The identification of CD8^+^ IFN-γ^+^IL-2^−^ T-cells to conserved epitopes of NP, M1, and PB1 (6), and CD3^+^IFN-γ^+^ T-cells to peptides covering NP and M1 (5) associated with protection against natural infection provides the first step toward the validation of such a T-cell correlate of protection. However, in both these studies a quantitative relationship between the magnitude of the T-cell response and risk of symptomatic disease was not reported. Granzyme B expression and the ratio of IFN-γ:IL-10 are also suggested as candidates for a correlate of protection, particularly in the elders (137, 138). A T-cell correlate of protection, particularly a quantitative correlate remains elusive. One study correlated an IFN-γ-secreting response on ELISpot with vaccine efficacy suggesting a threshold frequency of 100 SFCs/million PBMCs induced by LAIV to be associated with 50% reduction in risk of seasonal influenza (139). However, this observation has not been repeated. Irrespective of whether a correlate is identified, a significant challenge is the development of a standardized assay that combines enumeration of virus-specific T-cells with the multitude of markers for T-cell function and cytotoxicity.

Although an immune correlate of protection may be an immune marker related to protection against disease or infection but not necessarily the immune mechanism conferring protection (140), the inability to measure airway and lung T_RM_ cells in large cohorts of individuals to correlate with protection is a major limitation in identifying T-cell correlates of protection. Experimental influenza challenge studies to identify surrogate markers in peripheral blood of influenza-specific T-cells collected by bronchoalveolar lavage might be one approach to measure mucosal T-cells, although they still do not account for lung T_RM_ cells. Gene-expression analysis of the development of lung T_RM_ cells may identify unique signatures in peripheral blood that could be used as surrogates of these populations. Equally, the multifactorial nature of cross-protective immunity to influenza including non-neutralizing antibodies and different functional subsets of T-cells may be an inherent limitation to identifying a single quantitative T-cell correlate of protection. Multi-parameter flow cytometry and time-of-flight cytometry in combination with integrative biomodeling of immunological readouts may be a more robust tool to map the multifactorial nature of human T-cell responses (141).

It is revealing to track the development and use of the hemagglutination-inhibition (HI) titer of antibodies, the traditional correlate of protection against infection used for vaccine licensure. An HI titer of >1:40 was associated with a 50% reduction in influenza infection following experimental challenge (142). Although in use for over 40 years, there is emerging evidence questioning the use of an absolute titer as a marker of protection, its validity in children and elders, and its specificity in household transmission settings (143–145). Thus, it is not unreasonable to predict that a T-cell correlate will need to be modified for different age groups, vaccine types, and clinical outcomes. It is also noteworthy that mechanisms and magnitude of responses protective in human experimental challenges may not translate to natural community-based transmission settings because of differences in viral load, use of lab-adapted strains, and route of infection.

## Conclusion

Influenza vaccination is the most cost-effective public health strategy to combat influenza, since its introduction over 50 years ago. Current influenza vaccines are less effective against the inevitable emergence of new influenza strains. Harnessing heterosubtypic immunity is necessary to develop future influenza vaccines protective against a broad range of antigenically variant influenza strains for both seasonal and pandemic influenza. The evidence from animal models for memory T-cells in mediating heterosubtypic protection has underpinned the development of T-cell-inducing vaccines for influenza. Epidemiological evidence for natural heterosubtypic immunity in populations exposed to pandemic influenza aligned with recent studies, which provided the first direct evidence for a protective role for memory CD8^+^ T-cells in humans, has added new thrust to the search for a cross-protective influenza vaccine. Despite growing knowledge of the functional quality of T-cells, their antigenic targets, and development of sensitive T-cell assays, we do not have a T-cell correlate of protection. Memory T-cells resident in the lungs and circulating T-cells imprinted with lung homing signals are important in mediating cross-protective immunity while at the same time they can help cause lung pathology. Yet, we do not quite understand how to deliver antigens to preferentially induce the right quality of cells in the respiratory tract to skew the balance toward protection over tissue destruction. There is a great need to translate mechanistic observations made in animal models to the more complex human immune system. Our growing understanding of immune mediators of heterosubtypic immunity and the technological advances in vaccine design provide a perfect opportunity to produce a new pipeline of T-cell-inducing cross-protective influenza vaccines.

## Author Contributions

SS conceptualised and wrote the manuscript.

## Conflict of Interest Statement

The author declares that the research was conducted in the absence of any commercial or financial relationships that could be construed as a potential conflict of interest.
